# Supplementary material for: Immunogenic Properties and Antigenic Similarity of Virus-like Particles Derived from Human Polyomaviruses
Source: Int J Mol Sci. 2023 Mar 3;24(5):4907. doi: 10.3390/ijms24054907 (PMC10003412; doi:10.3390/ijms24054907)
Supplement: Supplementary file 1 [file ijms-24-04907-s001.zip › ijms-2236597-supplementary.pdf]

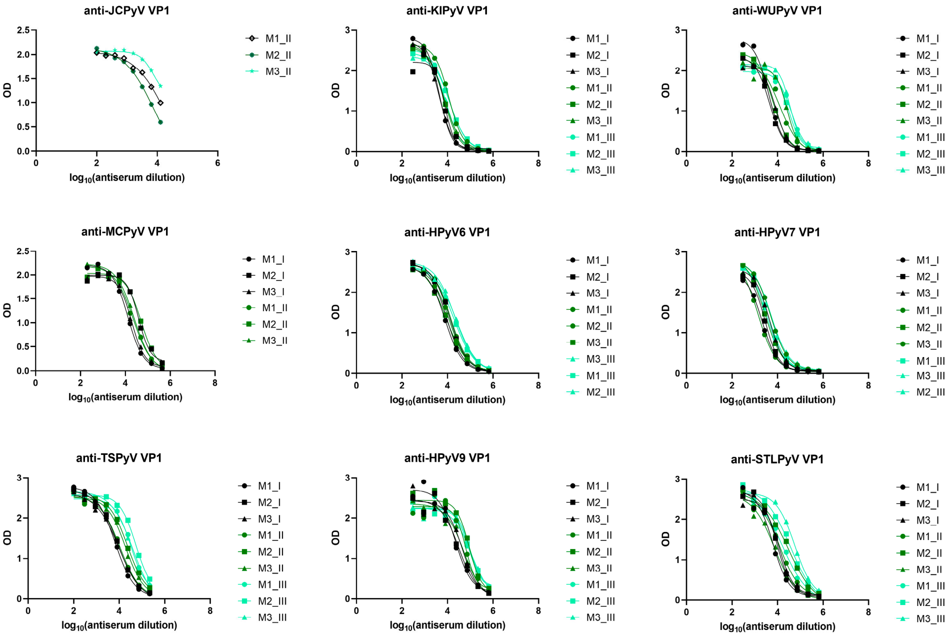

Figure S1. Titration curves of mouse antiserum after immunisation with VP1 VLPs of HPyVs. BALB/c mice (n = 3 per group) were immunised with recombinant PyV VLPs composed of VP1 protein. Antibody response against the PyV was determined by an indirect ELISA after the first, second, and third immunisation with or without an adjuvant (see method section). OD – optical density. M1, M2, and M3 represent mice in the group. I, II, and III represent the first, second, and third immunisation.

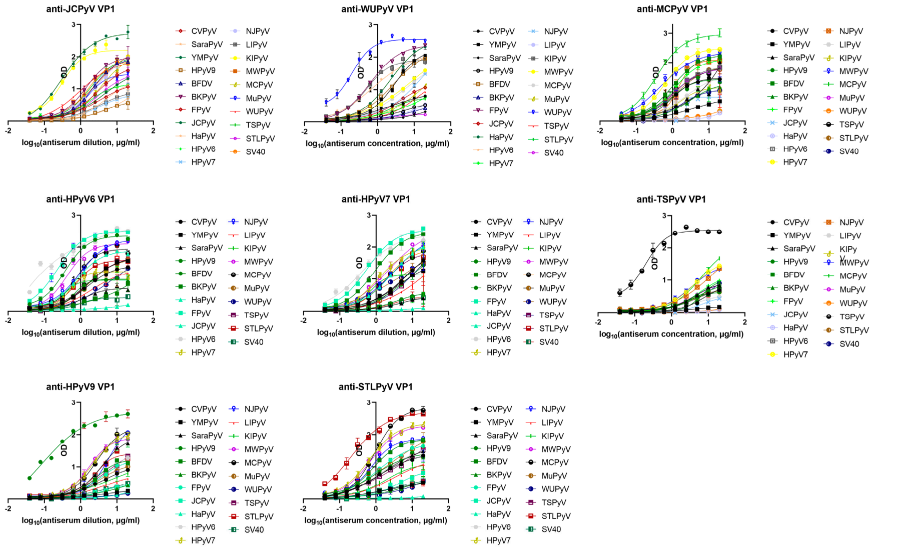

Figure S2. Titration curves of cross-reactivity of mouse antisera with VP1 proteins of different PyVs. Antisera raised against VP1-derived VLPs of different HPyVs were tested with different VP1 VLPs of human and animal PyVs by an indirect ELISA. The antiserum concentration (μg/mL) was equated to the total protein concentration. The diluent buffer was used as a negative control. OD: optical density.

Table S1. Titres of VP1-specific IgG in the antisera of mice immunised with VLPs of different PyVs. The values obtained from three immunized mice in each group are indicated. Titres which exceeded 1:15,000 are highlighted in red.

HPyV VP1 VLPs origin	Titre range			Size of VLPs, nm
	I immunisation	*II immunisation*	*III immunisation*	
JCPyV		7800–21,490		45–50
KIPyV	4300–7540	6800–11,410	8900–11,970	25–30
WUPyV	4130–8180	6250–25,640	28,260–39,430	25–35
MCPyV	12,960–38,960	22,110–50,240		40–45
HPyV6	7660–12,350	8730–13’500	19,490–23,790	25–35
HPyV7	2120–4760	1400–4980	3390–5120	25–40
TSPyV	6530–9480	10,750–22,840	316340–52,870	45–50
HPyV9	22,030–44,780	51,950–88,700	87,120–112,550	40–50
STLPyV	6230–11,710	7060–29,740	16,640–56,970	40–50

Table S2. Comparison of IgG titers raised against HPyV VP1-derived VLPs. IgG titres after the first, second, and third immunisation as one data unit was compared between different PyV VP1 VLPs. The IgG titres were determined by an indirect ELISA against the antigens used for immunisations. Significantly different pairs were highlighted. Pairs with HPyV9 VP1 VLPs are shown in red. ns: not significantly different, * $p < 0.05$, ** $p < 0.01$, *** $p < 0.001$, **** $p < 0.0001$ according to two-way ANOVA followed by Šídák’s multiple comparisons test.

Comparison pair of VP1 VLPs	Significance level	Comparison pair of VP1 VLPs	Significance level
JCPyV vs. KIPyV	ns	WUPyV vs. TSPyV	ns
JCPyV vs. WUPyV	ns	WUPyV vs. HPyV9	****
JCPyV vs. MCPyV	ns	WUPyV vs. STLPyV	ns
JCPyV vs. HPyV6	ns	MCPyV vs. HPyV6	ns, p = 0.06
JCPyV vs. HPyV7	ns	MCPyV vs. HPyV7	***
JCPyV vs. TSPyV	ns	MCPyV vs. TSPyV	ns
JCPyV vs. HPyV9	****	MCPyV vs. HPyV9	***
JCPyV vs. STLPyV	ns	MCPyV vs. STLPyV	ns
KIPyV vs. WUPyV	ns	HPyV6 vs. HPyV7	ns
KIPyV vs. MCPyV	**	HPyV6 vs. TSPyV	ns
KIPyV vs. HPyV6	ns	HPyV6 vs. HPyV9	****
KIPyV vs. HPyV7	ns	HPyV6 vs. STLPyV	ns
KIPyV vs. TSPyV	ns	HPyV7 vs. TSPyV	*
KIPyV vs. HPyV9	****	HPyV7 vs. HPyV9	****
KIPyV vs. STLPyV	ns	HPyV7 vs. STLPyV	ns
WUPyV vs. MCPyV	ns	TSPyV vs. HPyV9	****
WUPyV vs. HPyV6	ns	TSPyV vs. STLPyV	ns
WUPyV vs. HPyV7	ns	HPyV9 vs. STLPyV	****

Table S3. Monoclonal antibodies (MAbs) raised against VP1 VLPs of different PyVs. The clones of MAbs are indicated. The reactivity of the MAbs with the respective VP1 proteins were investigated by ELISA and WB.

	MAb Clone	Isotype				MAb Clone	Isotype		
VP1 VLPs			*ELISA*	*WB*	*VP1 VLPs*			*ELISA*	*WB*
JCPyV	2E4	IgG1	+	-	***HPyV7***	1H1	IgG1	+	+
	8E8	IgG2a	+	+		10E1	IgG2a	+	+
	8G8	IgG1	+	+		11A2	IgG1	+	+
	11C8	IgG2a	+	+		15A5	IgG1	+	+
WUPyV	2H3	IgG1	+	+		17H6	IgG1	+	+
	4E12	IgG2a	+	+		20F10	IgG1	+	+
	5H10	IgG2b	+	+		24B1	IgG2a	+	+
	11D2	IgG1	+	-	***TSPyV***	2B4	IgG2a	+	-
	11F5	IgG1	+	+		5E6	IgG2a	+	-
	11G10	IgG1	+	+		7A2	IgG1	+	-
	11G2	IgG1	+	-		9F2	IgG1	+	-
	12F8	IgG2a	+	+		9D3	IgG1	+	-
	12F1	IgG2a	+	+		16H6	IgG2a	+	-
KIPyV	5G8	IgG2a	+	-		17G11	IgG2a	+	-
	8D3	IgG2a	+	+		18A7	IgG2a	+	-
MCPyV	9G6	IgG1	+	-		19E4	IgG1	+	-
	24D11	IgG1	+	-		20D3	IgG1	+	-
	11A2	IgG2a	+	+	***HPyV9***	1F5	IgG2a	+	-
HPyV6	3A10	IgG1	+	+		5C4	IgG1	+	-
	6A9	IgG1	+	-		7E2	IgG1	+	-
	7B6	IgG1	+	-		8A10	IgG1	+	-
	12H1	IgG1	+	-		15B11	IgG2b	+	-
	15C11	IgG1	+	-		15B9	IgG1	+	-
	19C12	IgG2b	+	+		18G5	IgG2a	+	-
	23E1	IgG1	+	+	***STLPyV***	11A4	IgG1	+	-
	25F3	IgG1	+	-		14A12	IgG2a	+	-
	25D8	IgG1	+	+					

"+"—positive signal and "-"—no signal.
